# Induced perpendicular magnetization in a Cu layer inserted between Co and Pt layers revealed by x-ray magnetic circular dichroism

**DOI:** 10.1038/srep46132

**Published:** 2017-04-13

**Authors:** Jun Okabayashi, Tomohiro Koyama, Motohiro Suzuki, Masahito Tsujikawa, Masafumi Shirai, Daichi Chiba

**Affiliations:** 1 Research Center for Spectrochemistry, The University of Tokyo, 113-0033 Tokyo, Japan; 2 Department of Applied Physics, The University of Tokyo, 113-8656 Tokyo, Japan; 3 Japan Synchrotron Radiation Research Institute (JASRI), Kouto, Sayo, Hyogo 679-5198, Japan; 4 Research Institute of Electrical Communication, Tohoku University, Sendai 980-8577, Japan; 5 Center for Spintronics Research Network, Tohoku University, Sendai 980-8577, Japan

## Abstract

We used x-ray absorption spectroscopy and x-ray magnetic circular dichroism to investigate the effects of inserting Cu into Co/Pt interfaces, and found that a 0.4-nm-thick inserted Cu layer showed perpendicularly magnetized properties induced by the proximity effect through the Co and Pt layers. The dependence of the magnetic properties on the thickness of the Cu layers showed that the proximity effects between Co and Pt with perpendicular magnetic anisotropy can be prevented by the insertion of a Cu layer with a nominal threshold thickness of 0.7 nm. Element-specific magnetization curves were also obtained, demonstrating that the out-of-plane magnetization is induced in the Cu layers of the Co/Cu/Pt structures.

Perpendicular magnetic anisotropy (PMA) in magnetic multilayers with a high anisotropy constant (*K* [J · m^−3^]) is strongly desired for the development of high-density magnetic recording media because large PMA values can overcome thermal energy (*k*_B_*T*) near room temperature, where *k*_B_*T* ≈ 4 × 10^−21^ J as long as *KV/k*_B_*T* ≥ 60, even with nanoscale volumes (*V*) on the order of 10^−24^ m^3^ 1,2. CoPt alloys such as granular alloys and core-shell particles are candidates for demonstrating PMA through the large spin-orbit interaction of Pt atoms and the spin magnetic moments of Co atoms, which collaborate to enhance the PMA. There are many reports discussing PMA in Co/Pt interfaces, Co-Pt alloys, and granular thin films^3^,^4^,^5^,^6^,^7^,^8^, especially using x-ray magnetic circular dichroism (XMCD) to deduce the element-specific spin and orbital magnetic moments. Anisotropy of orbital magnetic moments is expected to play an important role in PMA. Recently, ultrathin Co/Pt layers have been extensively studied for the electric-field modulations of magnetic properties and spin-torque magnetic switching^9^,^10^,^11^. Moreover, the Co/Cu multilayer structures possessing in-plane anisotropy derived from the shape anisotropy in thin films have been investigated extensively because they are utilized for giant magneto-resistance devices^12^,^13^,^14^,^15^. The non-magnetic Cu spacer layers cause behavior similar to Ruderman-Kittel-Kasuya-Yosida (RKKY) oscillatory behavior in the exchange coupling between the magnetic Co layers^15^,^16^,^17^,^18^. Induced magnetism in Cu is also observed through the proximity effect with magnetic atoms^19^,^20^,^21^,^22^,^23^. Considering all of these physical phenomena, our present research had two goals: (i) the insertion of the Cu layer as an intermediate layer between Co and Pt in order to separate the magnetic coupling between them, and (ii) the attainment of induced PMA in Cu sandwiched between Co and Pt. To achieve these properties, we considered stacked structures of Co/Cu/Pt thin films with different Cu layer thicknesses.

There have been some prior efforts to insert a Cu layer into Co/Pt interfaces to prevent inter-diffusion at the interfaces and the resulting decrease of the PMA^24^,^25^. However, the problem of understanding the element-specific analysis in these structures remains unsolved. XMCD is a powerful technique to elucidate element-specific magnetic properties and to deduce the spin and orbital magnetic moments using the magneto-optical sum rules for each element^26^. Furthermore, it is well-accepted that PMA is proportional to the anisotropy of the orbital magnetic moments, as predicted by Bruno^27^. However, considering that the large spin-orbit interaction in Pt 5*d* states affects the PMA in the Co layer, the details of how to apply this formula are still being debated^28^,^29^,^30^,^31^,^32^, and the relationship between orbital magnetic moments and PMA is not completely understood. Therefore, a better understanding of the orbital moment anisotropy in the Co/Pt interface and its modulation by Cu insertion is required in order to implement novel material designs possessing PMA. Furthermore, the phenomena of induced PMA in non-magnetic elements have high potential for opening up a new research field considering the interfacial proximity effects. We emphasize that the detailed investigations for proximity-driven PMA using XMCD and theoretical calculation are the challenging subjects in spintronics research fields. In this paper, we investigate the element-specific spin and orbital moments of Co, Cu, and Pt in Co/Cu/Pt stacked structures using XMCD with element-specific magnetization curves in order to elucidate the origin of the proximity-effect-driven out-of-plane magnetization in the Cu inserted layer.

## Results

### Measurements of total magnetic moments

**Figure 1:**
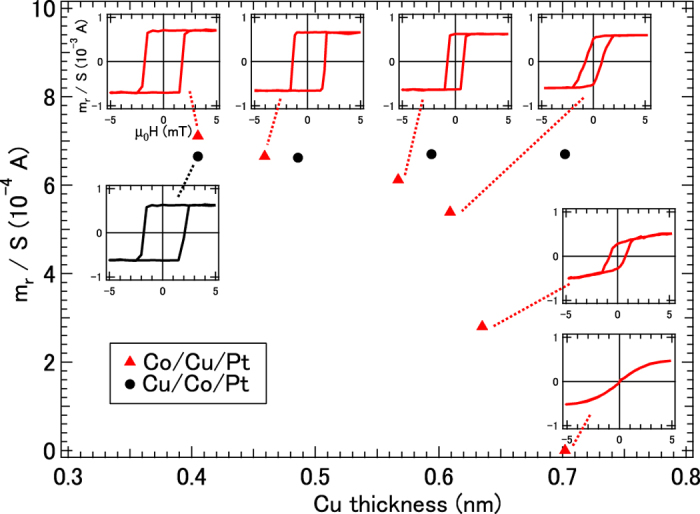
Dependence of the remanent magnetic moment *m*_r_ (per unit area *S*) on the Cu thickness for Co/Cu/Pt structures (triangles) and for Cu/Co/Pt structures (circles); the thicknesses of the Co and Pt layers were fixed at 0.4 and 2.0 nm, respectively, in all cases. The magnetic field dependence (*M*–*H* curves) for each value of Cu thickness are also shown in the case of the Co/Cu/Pt structures.

Figure 1 shows the dependence of the remanent magnetic moment of the stacked samples as a function of the thickness of the Cu layer. Since the individual contributions of the induced magnetic moments in the Cu and Pt layers are not separated, we adopt the units of Amperes, where the remanent magnetic moment (*m*_r_ [A · m^2^]) is divided by the sample area (*S* [m^2^]) for the ordinate of Fig. 1. For reference purposes, the cases in which the Cu layers were deposited on top of the Co/Pt layers are also displayed. The values of *m*_r_*/S* plotted in Fig. 1 for the Cu/Co/Pt structures are nearly constant, which demonstrates that the proximity effect between the Co and the Pt layers has a major influence on the PMA. However, when the Cu layer is inserted between the Co and Pt layers, *m*_r_ decreases rapidly as soon as the Cu thickness passes the 0.6 nm, suggesting that the proximity effects for PMA are weakened by the insertion of Cu layers. For a Cu thicknesses less than 0.6 nm, the *m*_r_ decreases slightly, and the line shapes in the hysteresis curves retain square features. Near the threshold thickness of 0.7 nm, the square shapes start to become broad, and *m*_r_ decreases as shown in Fig. 1. For Cu layer thicknesses larger than the threshold, the in-plane magnetic moment of Co derived from the demagnetization field in the films becomes dominant. These systematic changes suggest the disconnection between Co and Pt layer at the nominal threshold thickness of Cu 0.7 nm.

### XMCD study

**Figure 2:**
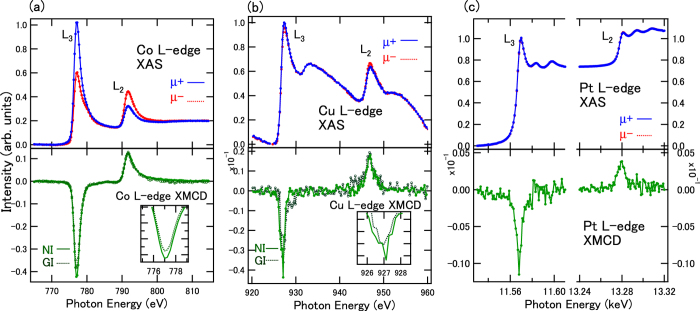
XAS and XMCD in Co (0.4 nm)/Cu (0.4 nm)/Pt (2 nm) structures for (**a**) Co, (**b**) Cu, and (**c**) Pt *L*-edges. XAS were taken at the NI setup. XMCD were taken at both NI and GI setups for Co and Cu *L*-edges, and at the NI setup for Pt. μ+ and μ− denote the absorption depending on the magnetic field directions. Insets show the expanded view of *L*_3_-edge XMCD signals.

Figure 2 shows the X-ray absorption spectroscopy (XAS) spectra and the XMCD spectra of Co, Cu, and Pt for Co/Cu/Pt structures with nominal thicknesses of 0.4, 0.4, and 2.0 nm, respectively, with surface capping layer of 2-nm-thick MgO. All XAS spectra were normalized at *L*_3_ edge intensities in order to compare the intensities of XMCD. In the Co *L*-edge spectra, clear metallic XAS line shapes and clear XMCD can be observed, suggesting that the Co atoms are not oxidized at the interface with the MgO capping layer. Asymmetry between *L*_3_ and *L*_2_ XMCD intensities brings the orbital magnetic moments within the framework of magneto-optical sum rules^26^. Angular dependence shows the difference in the intensity ratio. In the grazing incidence (GI) case, *L*_3_ intensities become smaller than those in the normal incidence (NI) case, suggesting that the orbital moment in GI is smaller than that in NI. On the other hand, extremely small XMCD intensities in the Cu *L*-edges were detected as shown in Fig. 2(b), which suggests that the XMCD signals in Cu are induced by the magnetic Co atoms with sign directions corresponding to the parallel alignments. Broad satellite structures in Cu *L*_2,3_-edge XAS are also observed at 931 eV and 952 eV corresponding to the transition from 2*p* to 4 *s* states, but these do not contribute to the XMCD signals. Similar satellite structures have been previously observed in the XAS spectra of Co/Cu multilayers^22^. In the case of Cu XMCD, very small differences in the intensity ratio between *L*_3_ and *L*_2_ can be observed, which suggests a small anisotropic orbital magnetic moment in Cu. In the Pt *L*_2,3_-edge XAS shown in Fig. 2(c), oscillatory line shapes appear above the main absorption edges due to the onset of extended X-ray absorption fine structure oscillation. The XMCD intensities in Pt *L* edges are weakened to half of maximum compared with those in the interface of Co (0.4 nm)/Pt (2.0 nm). This fact can be mainly explained by the insertion of Cu layers between Co and Pt because only the interfacial regions of Pt contribute to the magnetic properties through proximity with Co and Cu.

**Figure 3:**
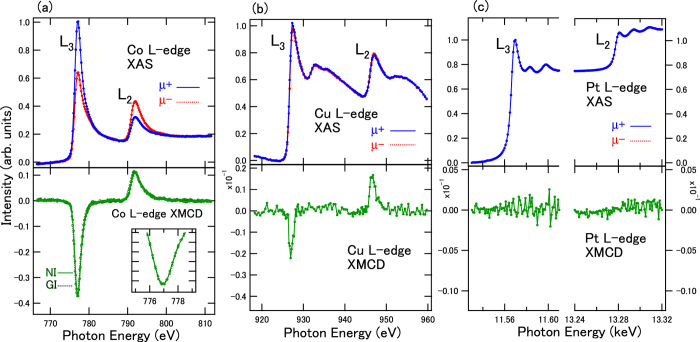
XAS and XMCD in Co (0.4 nm)/Cu (2.0 nm)/Pt (2 nm) structures for (**a**) Co, (**b**) Cu, and (**c**) Pt *L*-edges. XAS were taken at the NI setup. XMCD were taken at both NI and GI setups for Co *L*-edges, and at the NI setup for both Cu and Pt *L*-edges. μ+ and μ− denote the absorption depending on the magnetic field directions. Inset shows the expanded view of *L*_3_-edge XMCD signals.

Next, we show the XAS and XMCD in the case of 2-nm-thick Cu sandwiched between 0.4-nm-thick Co and 2-nm-thick Pt layers (Fig. 3). This sample shows in-plane magnetization. All XAS spectra displayed are also normalized in the same manner as shown in Fig. 2 in order to compare the XMCD signal intensities. In Co *L*-edge XMCD, angular dependence remains unchanged in the *L*_3_ and *L*_2_ intensity ratio, which suggests that the difference in the orbital moments in the case of in-plane anisotropy is suppressed through the disconnection between Pt and Co layers by the 2-nm-thick Cu layer. The Cu *L*-edge XMCD intensities become almost half of those in the 0.4 nm case shown in Fig. 2. This is derived from the fact that the interfacial Cu layer is spin-polarized and the bulk component in the 2-nm-thick Cu layer does not contribute to the spin polarization. Furthermore, the Pt *L*-edge XMCD signals cannot be observed as shown in Fig. 3 (c). These results indicate that the intermixing between the layers is suppressed, and the Co and Pt layers are disconnected.

Through spectral analysis of XMCD, we deduced the spin and orbital magnetic moments for each element, which are listed in Table 1. The effective spin moments including the magnetic dipole terms were estimated from the spin sum rule at NI and the spin moments were deduced from the analysis of the GI case^33^. The magnetic dipole terms were derived from the difference between these two values. The spin moment values for each element were calculated to be 1.67, 0.05, and 0.07 μ_B_ for Co, Cu, and Pt, respectively, with uncertainties of ±20%. We adopted the hole numbers of *n*_Co_ = 2.4, *n*_Cu_ = 0.4, and *n*_Pt_ = 1.8 which are estimated from the band-structure calculations. Suzuki *et al*. reported the spin and orbital moment values of Pt as 0.144 and 0.018 μ_B_, respectively, for the Co/Pt interface^8^. Both spin and orbital moment values of Pt deduced in our case are half of those estimated for the Co/Pt interface^4^,^8^ because of the suppression of the direct proximity between Co and Pt atoms. The perpendicular components in orbital moments of each element taken at NI were calculated to be 0.15, 0.01, and 0.005 μ_B_ for Co, Cu, and Pt, respectively. The values in Co and Cu are slightly larger than those reported previously for in-plane Co/Cu multilayers^17^. Although the interfacial Pt layer is spin-polarized by proximity, the polarization decays rapidly in the bulk. Since XMCD analyses include both interfacial and bulk contributions, we cannot deduce the precise values for Pt. However, we compared with the case of the same system without the Cu layer insertion. XMCD intensity in Pt becomes twice as large as the results in Fig. 2. Therefore, we confirmed not only the decay in bulk components but also proximity at the interfaces. The anisotropy of orbital moments Δ*m*_orb_ = (*m*_orb_^⊥^ − *m*_orb_^//^) in Co sites was calculated and found to have a value of 0.02 μ_B_. This quantity can be related to the PMA energy *K* arising from Co atoms by applying Bruno’s relationship: *K* = −(*G/H) ξ*_Co_Δ*m*_orb_/(4 μ_B_), where *ξ*_Co_ is the spin-orbit coupling constant of Co, and *G/H* is a parameter depending on the band structure^27^. Since the setup in the GI case involved a 60° angle between the magnetic field and the sample surface normal directions, the precise in-plane *m*_orb_^*//*^ cannot be detected; this underestimates the in-plane components. From the above results, we conclude that the interfacial *K* values are less than the order of 10^2^ μJ/m^2^ for Co sites. The anomalous Hall effect measurements represent the *K* value to be almost 10^2^ μJ/m^2^^34^,^35^. Therefore, orbital moment anisotropy for PMA can be adopted to Co sites where the large spin-orbit interactions of Pt atoms are responsible for PMA.

**Figure 4:**
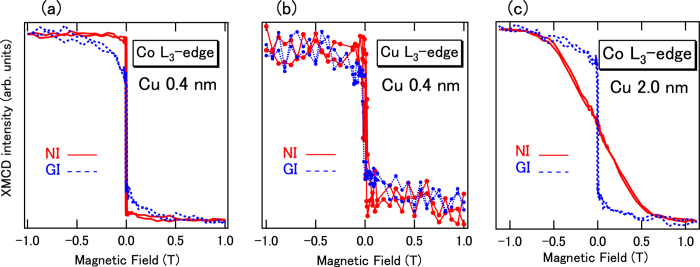
Element-specific magnetization curves, (**a**) Co (**b**) Cu for Cu 0.4-nm-thck Co (0.4 nm)/Cu (0.4 nm)/Pt (2 nm) structures and (**c**) Co *L*_3_-edges in Co (0.4 nm)/Cu (2.0 nm)/Pt (2 nm) structures. Both NI and GI directions are displayed.

Figure 4 shows the element-specific magnetization curves taken at photon energies of the Co and Cu *L*_3_-edges. In the case of the Co *L*_3_ edges, the angular dependence clearly displays the existence of PMA because GI x-rays show behavior typical of hard-axis directions, i.e., magnetic fields greater than ±0.5 T are necessary to achieve the saturation of the magnetization. When considering the magnetization curves of the Co *L*_3_-edge in the case of 2-nm-thick Cu as shown in Fig. 4(c), the magnetic easy axis in the Co sites is changed from the perpendicular to the in-plane direction with increasing Cu layer thickness. The features of the magnetization curves measured by XMCD are almost consistent with the measurements obtained by superconducting quantum interference device (SQUID) shown in Fig. 1. Here, we emphasize that the magnetization curve of Cu XMCD also displays out-of-plane magnetization as shown in Fig. 4(b), which is induced by proximity effects operating on both Co and Pt. In the case of Co/Cu multilayers, in-plane induced magnetization in Cu has been observed previously in XMCD^15^,^16^,^19^. Therefore, the out-of-plane magnetization in Cu is derived not only from Co but also from Pt spin-orbit interactions. Clear magnetization features in XMCD provide evidence that the out-of-plane magnetization in Cu in Co/Cu/Pt stacked structures originates from the contribution of all layers.

The XMCD results clearly suggest that the thick Cu layer prevents PMA at the Co/Pt interface, resulting in in-plane magnetism in Co and eliminating XMCD intensity in Pt because of the disconnection of the proximity effects at the Co/Pt interface. However, in the case of a thin Cu layer, of less than 0.7 nm, the proximity among Co, Cu, and Pt contributes to the PMA. Element-specific magnetization curves in XMCD successfully detected the out-of-plane magnetism in Cu.

### First-principles calculation

**Figure 5:**
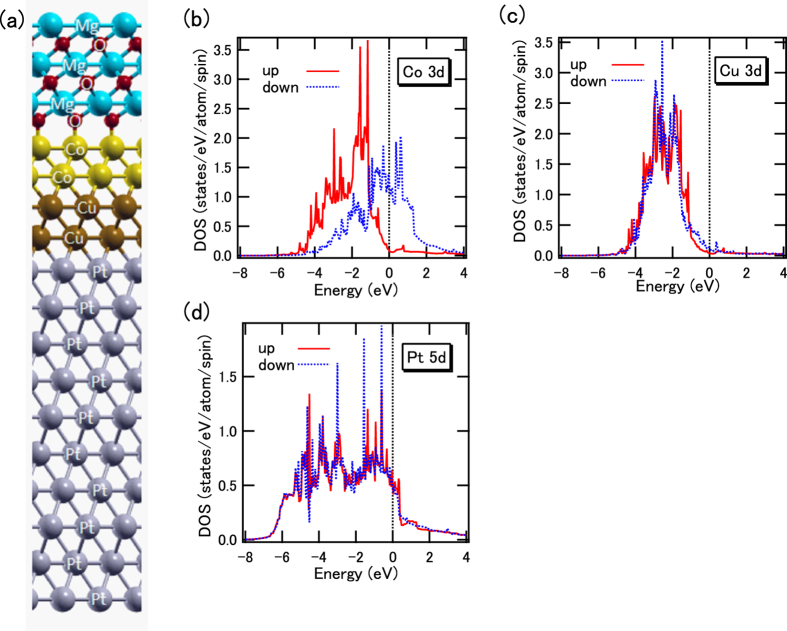
(**a**) Schematic stacked layers modeling for the first-principles calculation in MgO cap/Co 2 ML/Cu 2 ML/Pt 10 ML. Spin-resolved partial density of states for (**b**) Co 3*d,* (**c**) Cu 3*d*, and (**d**) Pt 5*d* states at the Co/Cu, Co/Cu and Cu/Pt interfacial layer, respectively.

We carried out a first-principles calculation for the MgO/Co/Cu/Pt stacked structure as shown in Fig. 5(a). We recognize the thickness of Co 2 monolayer (ML) corresponds to the 0.4 nm. Element-specific electronic structures of each layer can be deduced from the band-structure calculation. The density of states (DOS) of Co 3*d* states at the Co/Cu interface is clearly split with the spin magnetic moment of 1.72 μ_B_ as shown in Fig. 5(b). We also evaluated the magnetic anisotropy energy projected into each atomic site in order to analyze the origin of the PMA. Using the analysis for each layer, the orbital moment anisotropy of Co atoms at the Co/Cu interfacial layer was estimated to be 0.007 μ_B,_ and the PMA of the Co layers can be augmented mainly by the contribution of the interface layer between Co and MgO. The 3*d*_xz_ (*d*_yz_) orbital of Co atoms has a higher DOS around the Fermi level than the other 3*d* orbitals at the Co/MgO interface. Within the framework of second-order perturbation theory of the spin-orbit coupling^27^, the coupling between the *d*_xz_ and *d*_yz_ states above and below the Fermi level gives rise to the PMA. The DOS of Cu 3*d* states is also split as shown in Fig. 5(c) because of the proximity of the Co layers. Induced spin magnetic moments of 0.03 μ_B_ are also estimated in the Cu 3*d* states. However, induced orbital magnetic moments in Cu are estimated to be almost zero. Since the Cu 3*d* states are almost all occupied, the DOS at the Fermi level is quite small, which suggests that the Stoner conditions for ferromagnetism are not satisfied in Cu. The induced spin magnetic moment of Pt atoms at the Co/Pt interface was estimated to be 0.18 μ_B_, but it was decreased to 0.025 μ_B_ by inserting two-monolayer (ML) Cu because of the small spin splitting of Pt 5*d* DOS as shown in Fig. 5(d). The induced magnetic moments are caused by the *d*-orbital hybridization between neighbor Co and Pt atoms, which qualitatively coincides with the fact that the Pt XMCD intensities are suppressed by inserting Cu, in addition, the proximity effects are drastically suppressed by inserting Cu layer.

**Figure 6:**
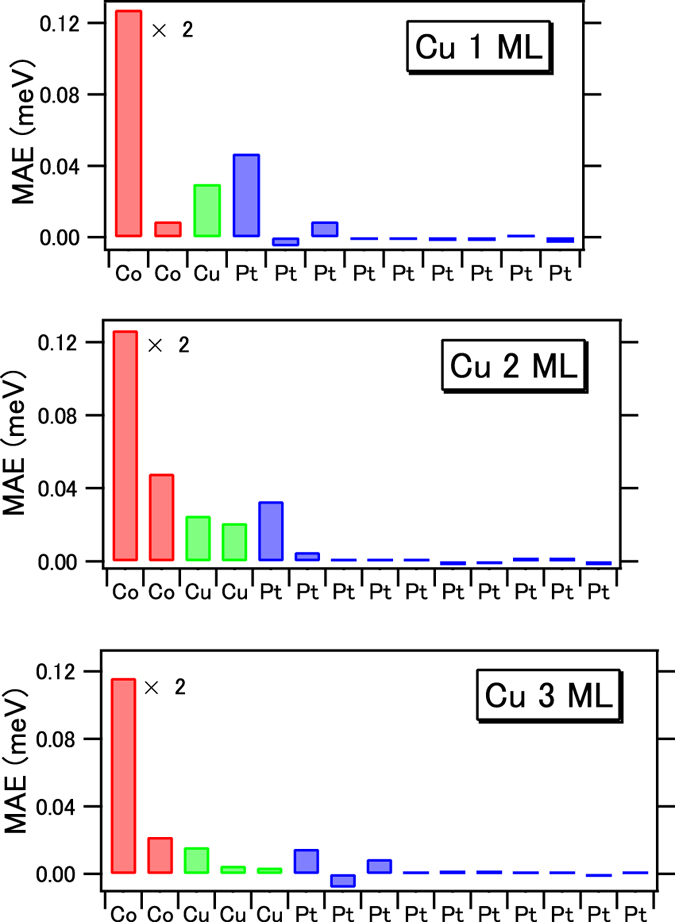
Layer-resolved magnetic anisotropy energy (MAE) depending on Cu layer thickness. Co layer thicknesses are fixed at 2 ML. The MAE of the top Co layer facing on MgO is twice as large as the plots.

In order to investigate the interfacial and bulk components, the magnetic anisotropy energy (MAE) in each layer is resolved and shown in Fig. 6. We performed the calculations for the Cu 1–3 ML cases. Positive MAE values stabilize the PMA. With increasing Cu layer thickness, PMAs in the Cu and Pt interfaces decrease. In the Cu 2 ML case, the Cu layers clearly contribute to the PMA. Note that the top Co shows a large PMA because of the contact with the artificial MgO interface. Furthermore, the lattice constants for Cu and Co are adjusted to that in Pt, which might induce strain effects for the magnetic layers. The theoretical calculations show the induced magnetism in Cu is derived from proximity with the Co layer, which supports the XMCD intensity in the Cu *L*-edges displayed in Fig. 2.

## Discussion

Considering the above results, we discuss the origins of out-of-plane magnetization in Cu. First, spin-orbit coupling constants *ξ* of Co and Pt are estimated by the first-principles calculations to be almost 60 and 550 meV/atom, respectively^29^. Since the orbital moment anisotropy in Co sites is derived from the spin-orbit interaction, the contribution of Pt atoms for the appearance of PMA becomes essential^30^. On the other hand, the exchange coupling between Co and Cu aligns the spin directions parallel to each other. As demonstrated in the Co/Cu multilayers with in-plane magnetization, RKKY-like oscillatory behaviors in the exchange coupling strength can be expected depending on the spacer-layer thicknesses^15^. Bruno’s relation using the orbital moment anisotropy is based on the assumption that the perturbation energy of the spin-orbit interaction is much weaker than the exchange splitting energy. The oscillatory behaviors of exchange coupling in the PMA cases might be stabilized by the competition between interfacial PMA energy *K* and exchange coupling constants, which introduces new physics to the modeling of the orbital magnetic moments. Interfacial PMA energy of Co sites is also estimated from the first-principles calculation. The calculation suggests an interfacial PMA energy of about 10^2^ μJ/cm^2^, on the same order as the results of the magnetization measurements and XMCD analysis. However, the calculation suggests that not only orbital moment anisotropy assuming the large exchange splitting but also the independent spin-flip contribution has to be treated at the interfaces in the MgO/Co/Cu/Pt system^32^.

Second, the relationship between proximity and exchange coupling in Cu must be discussed. PMA in Co/Pt is derived from the hybridization between Co 3*d* and Pt 5*d* wave functions, but that is disconnected by the Cu layer because the Cu 3*d* orbitals interrupt the Co-Pt proximity. Since the electronic structures of the three monolayers of Cu are different from those in the bulk, the exchange splitting in Cu can be induced through the hybridization with Co 3*d* states, which arises not from the Stoner conditions between para- and ferromagnetism but from proximity effects among Co/Cu/Pt layers. Therefore, the PMA energies in systems with inserted Cu are weakened.

Third, we comment on the comparison of spin magnetic moments as determined by the analysis of XMCD and by the band-structure calculation listed in Table 1. Band-structure calculation suggests that the magnetic moment values strongly depend on the layer structures. The subtle interfacial roughness affects the magnetic properties. The systematic Cu layer thickness dependence in the magnetization shown in Fig. 1 reveals the formation close to abrupt interfaces, which cannot be detected even by cross-sectional transmission electron microscopy for ultrathin films. Discrepancy in magnetic moments between the analysis of XMCD and the band-structure calculation might be derived from the interfacial roughness that is inevitably formed in the atomic scale, although physical pictures are reproduced qualitatively.

Fourth, we discuss the crystal structures of the thin Cu layer. The Fe/Cu interface showed body-centered-cubic, and the Co/Cu interface showed face-centered-cubic structures^21^. In both cases, induced XMCD in Cu layers was observed, suggesting that the proximity effects connected with ferromagnetic layers derive an induced magnetism in Cu that is independent of crystal structure.

Finally, we discuss the interfacial and bulk components in PMA. In the case of Cu 2 ML, both layers contact other elements. Although XMCD cannot distinguish these contributions, the layer-specific first-principle calculations depict the interfacial components. Although abrupt interfaces are assumed in the calculation, the qualitative tendency can explain the variation of XMCD results with Cu layer thicknesses.

In conclusion, we investigated the effect of inserting Cu into Co/Pt interfaces using XMCD along with the analysis of the element-specific spin and orbital magnetic moments in each element by means of magnetization curves in XMCD. We found that a 0.4-nm-thick inserted Cu layer shows perpendicularly magnetized properties that are induced by the proximity effect through the Co and Pt layers. Thickness dependencies of the Cu layers suggest that the proximity effects between Co and Pt with PMA can be prevented by insertion of a Cu layer with a nominal threshold thickness of 0.7 nm.

## Methods

Samples were prepared by RF sputtering. The stacked structures consisting of MgO (2.0 nm)/Co (0.4 nm)/Cu (*t* nm)/Pt (2.0 nm)/Ta (2.8 nm) layers were deposited at room temperature on Si (001) substrates (with the Ta next to the Si, and the MgO on top). The Pt layer is oriented normal to the [111] direction^34^. Here, the Cu layer thicknesses, *t*, of 0.4 and 2.0 nm were used for the PMA and for the in-plane anisotropy, respectively, in the XMCD measurements. The thicknesses of the layers were calibrated by the deposition rate of each material for the growth of precisely controlled stacked structures^34^. The MgO layer acted as a capping layer, preventing the oxidization of the sample surfaces. Magnetic properties of the samples were measured with a SQUID magnetometer.

XAS and XMCD measurements for Co and Cu *L*-edges were performed at BL-7A and 16A in the Photon Factory high-energy accelerator research organization (KEK) using the total electron yield mode. Photon helicity was fixed, and a magnetic field of ±1.2 T was applied parallel to the incident circularly polarized soft x-ray beam. For the Pt *L*-edge XMCD measurements, we employed the partial fluorescent yield mode at SPring-8 BL39XU^8^. All measurements were carried out at room temperature. Angular-dependent XMCD was performed by rotating the angle between the incident beam and the direction of the sample’s surface normal from the surface normal to 60°; these geometries are defined as NI and GI, respectively. In the case of the NI setup, where both the photon helicity and the magnetic field directions are normal to the surface, the X-ray absorption processes involve the normal direction components of the orbital angular momentum (*m*_orb_^⊥^). The GI setup mainly allows the detection of only the in-plane orbital momentum components (*m*_orb_^*//*^).

A first-principles band-structure calculation on the basis of density-functional theory was performed using the Vienna ab initio simulation package^36^,^37^ with the spin-polarized generalized gradient approximation (GGA)^38^. We constructed a supercell containing three layers of Mg, O, Co, and Cu atoms and five layers of Pt atoms. All atomic positions were relaxed until the Hellman-Feynman force was below 0.02 eV/Å with the in-plane lattice constant fixed to that of face-centered-cubic Pt (0.26 nm). The plane-wave cutoff energy was set to 500 eV and a 24 × 24 × 1 k-points Monkhorst-Pack mesh was used for sampling in the Brillouin zone. The magnetic anisotropy energy was evaluated using the magnetic force theorem. The atomic site projected magnetic anisotropy energy of *i* atom was calculated by the following equation:


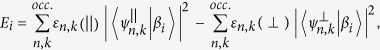


where *ε*_*n,k*_, *ψ*_*n,k*_ and *β*_*i*_ are the eigenvalue, the Bloch wave function with band index *n*, and the projector function on the atomic spheres, respectively.

## Additional Information

**How to cite this article**: Okabayashi, J. *et al*. Induced Perpendicular Magnetization in a Cu Layer Inserted between Co and Pt Layers Revealed by X-ray Magnetic Circular Dichroism. *Sci. Rep.* **7**, 46132; doi: 10.1038/srep46132 (2017).

**Publisher's note:** Springer Nature remains neutral with regard to jurisdictional claims in published maps and institutional affiliations.

## Figures and Tables

**Table 1:** Spin and orbital magnetic moments estimated from the XMCD sum rules for Cu thicknesses (*t*_Cu_) of 0.4 and 2 nm.

	Co		Cu	Pt
*t*_Cu_	0.4 nm	2 nm	0.4 nm	0.4 nm
*m*_orb_^⊥^ [μ_B_]	0.15 (0.108)	0.12	0.01 (0.002)	0.005 (0.003)
*m*_orb_^*//*^ [μ_B_]	0.12 (0.116)	0.12		
*m*_spin_ [μ_B_]	1.67 (1.72)	1.67	0.05 (0.03)	0.07 (0.03)
*m*_T_ [μ_B_]	0.02	0.01		

The in-plane (*m*_orb_^//^) and out-of-plane (*m*_orb_^⊥^) components are listed in units of μ_B_. The uncertainties for all values are estimated to be 20%. Values in parentheses are the spin and orbital magnetic moments of *d*-states estimated from the band-structure calculation for the Co, Cu and Pt atoms at the Co/Cu, Co/Cu and Cu/Pt interface, respectively.
